# The role and clinical value of natriuretic peptide receptor family in malignant tumor

**DOI:** 10.1038/s41420-025-02656-w

**Published:** 2025-08-28

**Authors:** Chuqi Quan, Weilin Shao, Yihao Yang, Qian Yao, Zuozhang Yang, Zhihong Yao

**Affiliations:** 1 Cancer Research Institute of Yunnan Province, Peking University Cancer Hospital Yunnan,Yunnan Cancer Hospital, The Third Affiliated Hospital of Kunming Medical University, Kunming, Yunnan China; 2 Bone and Soft Tissue Tumors Research Centre of Yunnan Province, Department of Orthopaedics, Peking University Cancer Hospital Yunnan,Yunnan Cancer Hospital, The Third Affiliated Hospital of Kunming Medical University, Kunming, Yunnan China

**Keywords:** Cancer, Tumour biomarkers

## Abstract

The natriuretic peptide receptor (NPR) family (NPRA, NPRB, NPRC) regulates diverse physiological and pathological processes. Mounting evidence implicates NPRs as key regulators of oncogenesis, metastasis, and therapy resistance in multiple cancers. This review integrates current understanding of the distinct mechanisms by which NPR members contribute to cancer development and progression, explores their molecular underpinnings, and discusses translational potential and future directions. A central focus is the context-dependent functional duality of NPR signaling, where specific subtypes act as either oncogenic drivers or tumor suppressors depending on the malignancy.

## FACTS

- NPRA acts as an oncogenic driver; its overexpression correlates with poor prognosis in breast, prostate, and gastric cancers.
- NPRA promotes angiogenesis via VEGF upregulation.
- NPRA knockdown suppresses tumor growth, metastasis, and angiogenesis in preclinical models.
- NPRB plays context-dependent roles in cancer development.
- NPRC functions primarily as a tumor suppressor.
- Therapeutic and diagnostic potential:
- NPRA is a prognostic biomarker in gastric/esophageal cancers.
- NPRC agonists (e.g., C-ANP4-23) show antitumor effects in preclinical models.

## OPEN QUESTIONS

1. How do NPs/NPRs crosstalk with oncogenic pathways (e.g., Wnt/β-catenin, JNK)?
2. Do NPs/NPRs influence radiotherapy, chemoresistance and immunotherapy response (e.g., anti-PD-1 resistance) in antitumor treatment?
3. Can NPR based biomarkers be integrated into liquid biopsy platforms for early detection?

## Introduction

The Natriuretic Peptide (NP) is well recognized for its wide range of biological activities. Apart from stabilizing the cardiovascular system, they have been implicated in immunity, inflammatory responses, and cancer [1–4]. The NP includes atrial natriuretic peptide (ANP), brain natriuretic peptide (BNP) and c-type natriuretic peptide (CNP). ANP was initially discovered in atrial cardiomyopathy extracts a diuretic-promoting substance found in atrial myocardial extracts [5]. BNP was obtained from porcine neural tissue, and in cardiomyocytes [6]. While CNP was initially extracted from pig brain. This peptide has also been detected through immunoreactivity in human vascular endothelial cells, as well as in renal, reproductive, and various other tissues [7]. The receptors for these natriuretic peptides comprise at least three isoforms, namely A-type (NPRA, GC-A), B-type (NPRB, GC-B) and C-type (NPRC) [8, 9]. NPRA and NPRB are guanylate cyclase-coupled receptors, activating cyclic GMP (cGMP) signaling, NPRC mainly serves as a receptor responsible for peptide clearance. Originally identified for their roles in cardiovascular regulation, the NPR family has recently attracted interest in cancer research due to its abnormal expression in various tumors. Their dysregulation in cancers underscores their potential as biomarkers and therapeutic targets. Modified NPs (e.g., long-acting chimeric peptides or fusion proteins) may become novel anticancer drugs, which is particularly relevant for tumors that do not respond to standard treatment methods [10]. Dysregulation of NPR expression across malignancies highlights their dual roles in tumor biology and positions them as promising biomarkers and therapeutic targets. This review comprehensively delineates the pathophysiological significance of NPRs in cancer progression, dissects their mechanistic interplay with oncogenic pathways, and critically appraises clinical translation strategies.

## Molecular structure and functional basis of the natriuretic peptide receptors

**Fig. 1:**
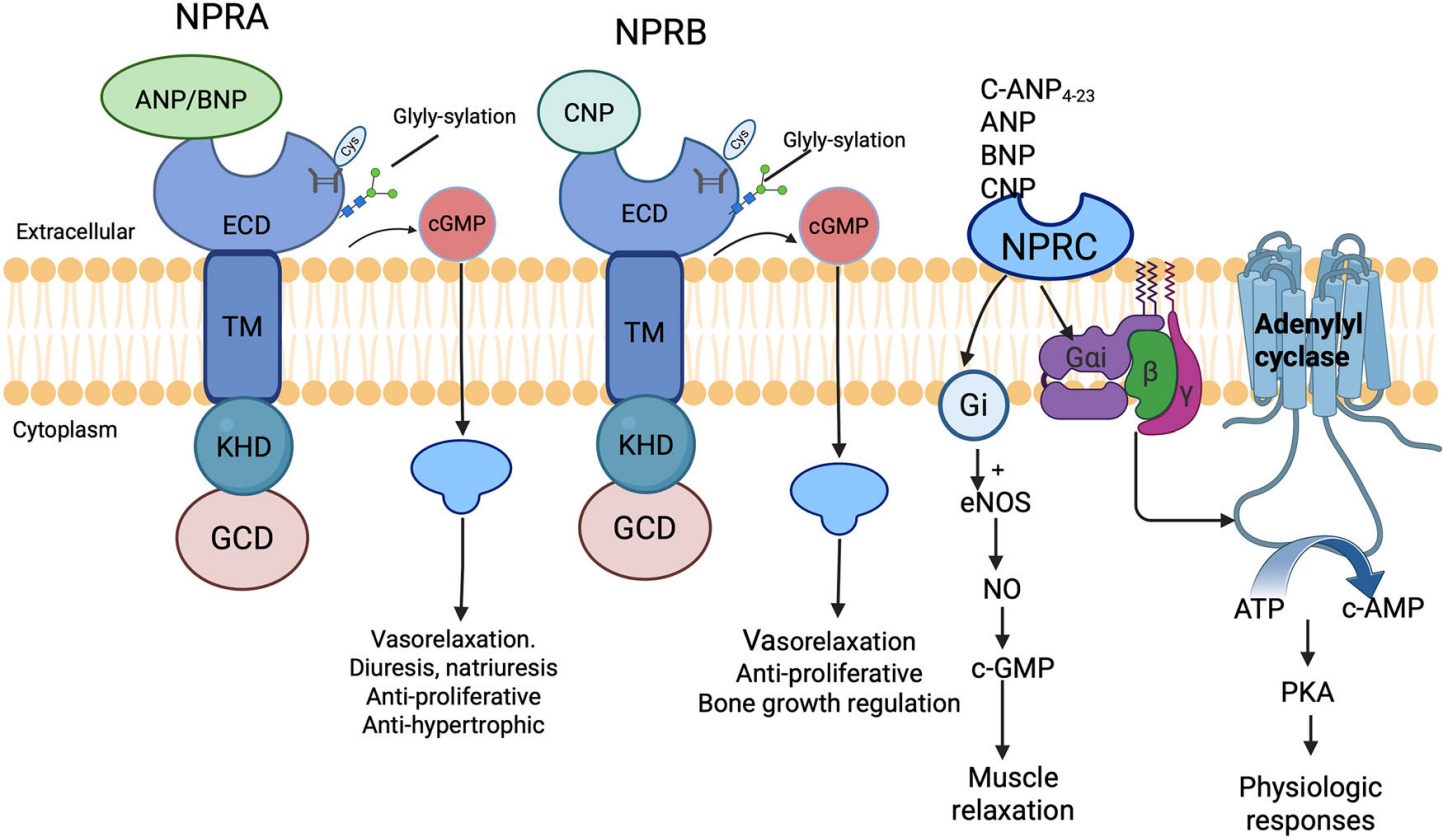
Overview of the structural domains and ligand binding of the NPR family. The natriuretic peptide receptor (NPR) family consists of three main members: NPRA (NPRA/GC-A), NPRB (NPRB/GC-B), and NPRC (NPRC). While NPRA and NPRB are transmembrane guanylate cyclase (GC)-coupled receptors, NPRC lacks intrinsic GC activity and primarily functions as a clearance receptor. The extracellular domain of NPRA binds ANP and BNP with high affinity, which contains disulfide bonds critical for ligand recognition. NPRB shares 70% sequence homology with NPRA but is preferentially activated by CNP. NPRC binds all NPs (ANP > BNP > CNP) with high affinity but lacks transmembrane and catalytic domains. NPRC competes with NPRA/B for ligand binding, reducing the bioavailability of NPs (e.g., ANP, BNP) and indirectly modulating NPRA/B signaling.

To provide an accessible conceptual framework, we have comprehensively integrated the structural architecture and functional underpinnings of the NPs/NPRs system into a schematic diagram (Fig. 1).

### NPRA

The NPRA gene is approximately 16 kb and contains 22 exons and 21 introns. All of the exon-intron junctions correspond to GT/AG consensus sequences. Sequences encoding the transmembrane structural domains (a portion of the NPRA topology) are present in exon 7 [11]. NPRA also referred to as particulate guanylate cyclase A, is a receptor with strong binding affinity for ANP as well as BNP. NPRA possesses a glycosylation-binding domain located on its extracellular region that is connected to a hydrophobic transmembrane segment, a kinase-like, non-catalytic structural domain that binds ATP, located near the cytosolic end of the molecule, which is believed to be involved in controlling receptor function, along with a brief hinge region. NPRA contains particulate guanylate cyclase, which is responsible for their catalytic activity. Upon binding to its specific ligands (ANP or BNP), NPRA experiences a structural shift in the kinase-like domain, leading to the release of guanylate cyclase inhibition and a subsequent increase in cGMP synthesis [12]. Cryo-electron microscopy investigations have revealed that NPRA is an ornithine homodimer composed of a central Broad-complex, Tramtrack, and Bric-à-brac (BTB) structural domain, along with a BTB domain and carboxy-terminal Kelch helical bundles, and that NPRA is extensively distributed in multiple tissues, such as blood vessels, the heart, kidneys, lungs, adrenal glands, and adipose tissue, testes, liver, and immune system, as well as in certain cancers. It is particularly prominent in blood vessels, heart tissue, renal structures, pulmonary tissues, and smooth muscle. From a structural perspective, NPRA is made up of four main functional regions: an extracellular ligand-binding domain (ECD), a single transmembrane region, an intracellular protein Kinase Homology Domain (KHD), and a guanylate cyclase catalytic domain (GCD) [13]. The ICD includes a protein kinase-like domain (PKLD) and a catalytic domain (GCD), with PKLD modulating activity via phosphorylation and ATP binding, and the GCD is responsible for the generation of cGMP. ANP binding leads to a 24° rotational movement of the ECD dimer, resulting in a relative displacement of the near-membrane structural domain. This conformational change is conveyed to the cell via the transmembrane helix, triggering activation of GCD for transmembrane signaling [14].

### NPRB

The NPRB gene is approximately 16.5 kbp, over 30-fold larger than cDNA. The exons vary in length from 69 bp (exon 18) to 667 bp (ATG-exon 1), while the introns span from 87 bp (intron 14) to 6.5 kbp (intron 3) [15]. NPRB serves as the primary receptor for natriuretic peptides within the brain. Besides, it is also a particulate guanylate cyclase-linked receptor that increases intracellular cGMP concentration upon receptor activation [9, 16**]**. CNP is an isolated agonist of NPRB [9]. The overall topology of NPRB is the same as NPRA, whose disulfide bond patterns and glycosylation sites have not yet been determined by chemical methods. However, studies utilizing mutagenesis techniques have revealed the presence of intramolecular disulfide bonds connecting Cys-53 to Cys-79, Cys-205 to Cys-314, and Cys-417 to Cys-426. Additionally, findings indicate that five out of the seven extracellular asparagine residues undergo glycosylation [17, 18]. To date, no crystallographic structures have been reported for any specific domain within the architectural framework of NPRB. Furthermore, numerous alternative splice forms of NPRB have been identified, one of which is devoid of enzymatic activity and acts in a dominant-negative fashion [19, 20], Nevertheless, it is not yet fully understood whether these truncated isoforms contribute to CNP signaling. The arrangement of NPRB stimulation by natriuretic peptides occurs in the following sequence: CNP, ANP, and BNP. To date, there have been no published studies on purified NPRB.

### NPRC

The human NPRC gene extends over 65 kb and consists of eight exons and seven introns, which contain the GT/AG consensus linkage sequence [21]. Unlike other G-protein-coupled receptors, the NPRC features a solitary transmembrane segment accompanied by a brief cytoplasmic region made up of 37 amino acids, distinguishing it from other receptors with a single transmembrane domain. This structure is distinct from other receptors with a single transmembrane domain. This cytoplasmic peptide, consisting of 37 amino acids, effectively inhibits adenylyl cyclase activity and harbors an epitope with a Ki comparable to ANP99-126 or C-ANP4-23. Moreover, C-ANP4-23 activated phosphatidylinositol (PI) conversion in Vascular Smooth Muscle Cells (VSMC). This effect was reduced by the presence of dbcAMP as well as cAMP activators. It is suggested that the suppression of adenylate cyclase activity by the NPRC, leading to a reduction of intracellular cAMP concentrations, could potentially explain the observed stimulation of PI conversion mediated by NPRC. Additionally, the stimulation of NPRC by C-ANP4-23 as well as CNP inhibited the mitogen-activated protein kinase activity triggered by endothelin-3, platelet-secreted growth factor, and a synthetic lipid compound used to activate protein kinase C (PKC). This suggests the NPRC receptors could be linked to additional signaling pathways or that there may be crosstalk between NPRC receptors and other signaling mechanisms [22].

## Dual regulatory roles of natriuretic peptide receptors in tumorigenesis

NPRA is overexpressed in multiple cancers, activating oncogenic pathways. NPRB regulates tumor vascular normalization. It was shown that engineered CNP (dCNP) specifically activating NPRB improves vascular structure/function. dCNP enhances endothelial cell junctions and pericyte coverage via cGMP, reducing leakage [23]. Contrasting NPRA, NPRC exerts tumor-suppressive effects (Table 1).

**Table 1:** Characteristics and functions of natriuretic peptide receptor family members.

Receptor type	Gene location	Primary ligands	Signaling pathway	Physiological function	Primary role in tumors
**NPRA (NPR1/GC-A)**	1q21.3	ANP > BNP > CNP	cGMP/PKG	Cardiovascular homeostasis, BP regulation	**Pro-tumor**: Promotes angiogenesis, inhibits apoptosis
**NPRB (NPR2/GC-B)**	9p13.3	CNP > ANP ≥ BNP	cGMP/PKG	Cartilage development, bone formation, heart rate regulation	**Dual-role**: Promotes vascular normalization, inhibits metastasis
**NPRC (NPR3)**	5p13.3	ANP > CNP > BNP	Clearance/Gi-protein	Peptide clearance, vascular tone regulation	**Antitumor**: Promotes apoptosis, inhibits proliferation

### NPRA and cancer

#### Breast cancer

In a study, researchers found that NPRA expression was elevated in breast cancer tissues, with higher levels of NPRA being linked to poor clinicopathological outcomes. Breast cancer patients who have an elevated level of NPRA expression tend to have a reduced chance of survival after 5 years, and thus NPRA may be an independent predictor of prognosis in individuals diagnosed with breast cancer [24]. Reducing NPRA expression reduces the growth, movement, and metastatic potential of tumorigenic cells from breast tissue, while increasing its expression enhances their malignant traits. In addition, evidence suggests that NPRA enhances invasiveness of malignant neoplasm cells by promoting the upregulation of matrix metalloproteinase-9 (MMP9), the mechanism of which may be that NPRA increases MMP9 expression through activation of STAT3 [24]. Studies have shown that NPRA may act as a predictor of prognosis, with p-STAT3 and MMP9 recognized as likely targets of NPRA in patients diagnosed with breast cancer [24]. In addition, the formation of new blood vessels within tumors is essential for the progression and metastasis of breast cancer cells. Mao et al. [25] reported that glipizide, a medication commonly prescribed for treating type 2 diabetes mellitus, inhibited tumor growth and metastasis. Their study revealed that the combination of glipizide and ANP effectively suppressed the development and spread of breast cancer in MMTV-PyMT mice, which naturally developed breast tumor. This study confirmed ANP in combination with glipizide was more effective than glipizide alone to be repurposed as an effective agent for the treatment of breast cancer by targeting tumor-induced angiogenesis.

#### Gastric cancer (GC)

NPRA plays a critical role in facilitating GC development as well as disease progression. NPRA is highly present in malignant gastric tumor tissues and positively correlates with the vascular marker CD31 and vascular density. The high expression of NPRA predicts that patients with gastric cancer have a poor prognosis. Studies conducted under controlled laboratory and tissue-based conditions have demonstrated that NPRA facilitates the advancement of gastric cancer by enhancing angiogenesis and promoting metastasis. Li et al. [26] found that NPRA directly binds to HIF-1α as well as prevents its degradation by the ubiquitin-proteasome system by Co-IP/MS and immunoprecipitation experiments, thereby stabilizing the protein level of HIF-1α.Stabilization of HIF-1α increase leads to upregulation of downstream VEGF (vascular endothelial growth factor) expression, which in turn promotes angiogenesis. Whereas knockdown of NPRA reduced VEGF secretion and inhibited the proliferation, tube formation, mobility and capacity for invasion of vascular endothelial cells (HUVEC). Overexpression of HIF-1α reversed this effect. In addition, NPRA has the potential to represent a potential new avenue for therapeutic intervention in gastric malignancies, and combined inhibition of NPRA and autophagy enhances the antitumor effect. Immunohistochemical analysis demonstrated that elevated levels of NPRA expression are observed in the case of gastric malignancies, showed a positive association with the tumor’s size and progression stage. Li et al. [27] found that knockdown of NPRA resulted in G2/M-phase arrest in gastric cancer cells (SGC-7901 and BGC-823), which was associated with the downregulation of Cyclin B1 and Cdc2 expression. NPRA inhibited the induction of apoptosis and necrosis and enhanced cell death through caspase-dependent pathways. Cell death NPRA knockdown activates autophagy through ROS accumulation, but autophagy contributes a protective function during this mechanism. Inhibition of autophagy further exacerbates cell death. NPRA inhibition also leads to mitochondrial damage and ROS accumulation. These results propose that NPRA could facilitate the advancement of gastric cancer. Zhang et al. [28] reported that NPRA was highly expressed in AGS of gastric cancer cells. They found that ANP directly regulated the activation characteristics of potassium currents by membrane clamp experiments. Western blot and qPCR confirmed that KCNQ1 expression, the levels of expression, detected at both mRNA and protein stages, were regulated in a bidirectional manner depending on the concentration of ANP. Lower levels of ANP promoted the growth of AGS cells, increased KCNQ1 expression, and boosted potassium currents sensitive to TEA and 293B, whereas higher concentrations of ANP decreased AGS cell proliferation, down-regulated KCNQ1 expression, and reduced potassium currents. These data suggest that the concentration-dependent bidirectional regulatory effect of ANP on gastric cancer cell proliferation is driven by KCNQ1-mediated changes in potassium channel activity, suggesting that KCNQ1 shows potential as an effective therapeutic target in the treatment of gastric cancer. NPRA further enhances the stemness and chemoresistance of cancer cells in gastric cancer by promoting fatty acid oxidation via the MSC-NPRA feedback loop. Gastric cancer patients often have a poor prognosis due to cisplatin (CDDP) chemoresistance. Mesenchymal stem cells (MSCs) have been shown to be involved in chemoresistance in the tumor microenvironment. Chen et al. [29]. found that MSCs promote stemness and CDDP resistance by up-regulating NPRA within gastric cancerous cells through the process of MSCs up-regulating NPRA in gastric cancer cells via secreted factors (e.g., ANP). And NPRA, in turn, promotes the recruitment of MSCs into the tumor microenvironment. A sustained and enhanced pro-cancer cycle is formed. NPRA prevents the mitochondrial fusion protein Mfn2 from ubiquitination degradation by binding to and protecting it and promotes the localization of Mfn2 in the outer mitochondrial membrane. Stabilization of Mfn2 enhances mitochondrial function, which in turn activates fatty acid oxidation (FAO), as evidenced by the upregulation of CPT1 expression, reduction of lipid droplets, and increase of ATP production. Enhanced FAO maintains the mitochondrial function of the mitochondrial fusion protein Mfn2 by supplying ATP and metabolic intermediates, maintaining the expression of tumor stemness markers (e.g., SOX2, CD44) and inhibiting chemotherapy-induced apoptosis. This mechanism reveals the central role of the MSC-NPRA-Mfn2-FAO axis in chemoresistance of gastric cancer and offers a conceptual foundation for the advancement of therapeutic regimens targeting metabolism or NPRA [29]. Cao et al. [30] showed that NPRA promotes the cell division and expansion of gastric cancer cells by interacting with and stabilizing PPARα, and up-regulating CPT1B-mediated fatty acid oxidative metabolism.

#### Squamous cell carcinoma

Nakao et al. found [31] that the elevated expression of NPRA in tongue squamous cell carcinoma (TSCC). It was notably increased compared to normal oral epithelium and strongly correlated with tumors that exhibited high invasiveness. The overexpression of NPRA exhibited a significant relationship with the concentrations of VEGF-A, a pro-angiogenic factor, and the pro-lymphangiogenic factor, VEGF-C, suggesting that NPRA may contribute to the development of tumor vasculature and lymphangiogenesis by modulating the VEGF pathway, which in turn enhances invasion and metastasis. The study suggests that NPRA can be used a predictive biomarker and possible therapeutic target for TSCC. In esophageal squamous cells, the expression of NPRA protein is significantly higher than in normal tissues and is mainly localized in the cytoplasm [32]. The expression rate of NPRA was notably higher in cancer tissues compared to non-tumor tissues. Clinicopathological evaluation revealed a correlation between elevated NPRA expression and both tumor differentiation and TNM staging. However, it did not show any significant association with age, gender, or lymph node metastasis. Western blot showed that NPRA expression in ESCC cell lines (Eca109, TE-1) was significantly increased relative to the levels observed in normal esophageal epithelial cells (Het-1A). Transwell assays demonstrated a marked decrease in the migration and invasion of Eca109 cells following NPRA downregulation via sh-RNA, and NPRA may promote tumor invasion through activation of matrix metalloproteinases MMP2 and MMP9. NPRA may be a molecular marker of aggressive behavior in ESCC, suggesting the possibility of its use as a therapeutic target.

#### Prostate cancer

NPRA promotes prostate cancer progression by regulating inflammatory factors such as MIF. Wang et al. [1] illustrated that NPRA was significantly highly expressed in tumorigenic prostate cells (e.g., PC3, DU145, and TRAMP-C1), while it was barely expressed in normal PrEC and BPH cells, and NPRA expression was positively correlated with prostate cancer stage (e.g., Gleason score) and androgen-independent (AI) status. Down-regulation of NPRA by si-NPRA or inhibitors (iNPRA) induces apoptosis in prostate cells. Their study suggests that the mechanism by which iNPRA induces anti-PCa effects is related to NPRA-mediated upregulation of macrophage MIF. An overexpressed cytokine associated with inflammation in Prostate cancer, which is markedly decreased by siNPRA. Treatment with NPRA inhibitors, such as plasmid-encoded iNPRA NP73-102, significantly reduced the tumor load of mouse transplanted tumors and was accompanied by downregulation of NPRA and MIF expression. Therefore, strategies targeting NPRA may provide new directions for prostate cancer treatment. Vesely and colleagues [33] verified the influence exerted by four peptide hormones encoded through the expression of the ANP gene (Long-acting Natriuretic PAeptide, Vasodilator, Diuretic Peptide, and Atrial Natriuretic Peptide) on PCa, and found that all four peptide hormones dose-dependently inhibited prostate cancer cells at increasing concentrations (PC- 3 lineage), with vasodilator having the strongest effect, followed by ANP, kaliuretic peptide and LANP. Their study demonstrated for the first time that prostate cancer cells express NPRA and NPRC, suggesting that hormones act through the receptor-mediated signaling pathway, and providing experimental evidence for the development of novel prostate cancer targeted therapies based on the hormonally active peptides of the ANP gene, especially the effects of vasodilator and ANP, on prostate tumor cells. basis, especially potent effects of vasodilators deserve further exploration. In addition, they found that the four peptide hormones also play a role in pancreatic cancer. Through cGMP-mediated inhibition of DNA synthesis, they notably decreased the quantity of pancreatic adenocarcinoma cells and inhibited their proliferation, providing a new research direction for the treatment of highly lethal adenocarcinoma [34].

### NPRB and cancer

#### Prostate cancer

Guanylate cyclase B, alternatively referred to as NPRB, is one of the seven particulate guanylate cyclases isolated from mammalian tissues, besides, it was discovered that CNP serves as its endogenous ligand. It has been established that the CNP/GC-B/cGMP pathway is crucial for the local regulation of vascular and skeletal pathways, neurological and tissues associated with reproduction [35]. Whereas Lippert et al. [36] found that messenger RNA levels of CNP as well as NPRB in prostate tissue were significantly reduced as tumor progression advanced. Expression in seminal vesicle tissue was not affected by tumor stage. In contrast, CNP and pro-CNP concentrations in seminal plasma increased significantly with increasing tumor load. Thus, downregulation the expression of the local CNP system in the prostate may be associated with tumor progression, whereas elevated concentrations in seminal plasma may reflect the effect of the disease on the secretory function of the prostate.

### NPRC and cancer

#### Prostate cancer

Early diagnosis of prostate cancer lacks specific imaging probes. Research has shown that the NPRC exhibits elevated expression levels in prostate cancer cells. The research conducted by Pressly, E.D. and colleagues [37] demonstrated the expression levels of NPRC were verified in cancerous tissues, especially in inflammatory cells and vascular endothelial cells at the tumor margin. They were pioneers in identifying NPRC as a promising candidate for prostate cancer imaging applications, expanded application of the natriuretic peptide receptor in tumor diagnosis, and developed a nanoprobe targeting the NPRC receptor, which was demonstrated to be highly efficient and specific in PET imaging of prostate cancer through precise molecular design and in vivo validation, providing a new strategy for clinical translation.

#### Colorectal cancer (CRC)

Research has demonstrated that lncRNA BCYRN1 expression is notably elevated in CRC cells and extracellular matrices relative to the surrounding normal tissues. Gu et al. [38] found by microarray analysis and validation experiments that lncRNA BCYRN1 acts through the upregulation of NPRC, and that knockdown of lncRNA BCYRN1 contributed to a decrease in the levels of expression of NPRC, which itself has a pro-proliferative and anti-apoptotic function, thus, lncRNA BCYRN1 promotes proliferation and inhibits apoptosis of colorectal cancer cells by up-regulating NPRC. However, the article did not elucidate the specific molecular mechanism by which lncRNA BCYRN1 regulates NPRC. Another study by Jorge Martinez-Romero et al. [39] supported this conclusion, which analyzing a dataset of 1273 human colorectal cancer patients to screen for candidate target molecules associated with patient prognosis. They identified NPRC and nine other highly expressed molecules showing a strong correlation with colorectal cancer prognosis. However, this study also did not provide further in vitro or in vivo validation of the molecular mechanisms through which NPRC regulates colorectal cancer. Therefore, further in-depth research into these mechanisms remains limited, highlighting the need for subsequent validation studies. It would be highly valuable to conduct such validation to elucidate NPRC’s role in colorectal cancer.

#### Clear cell renal cell carcinoma (ccRCC)

The study reported by Li et al. [40] found that lncRAN MRCCAT1 epigenetically reduces the activity of NPRC transcription through the recruitment of PRC2 to the NPRC gene regulatory sequence, thereby enhancing the H3K27me3 modification the repression of NPRC leads to an elevated level of p38-MAPK phosphorylation and promotes tumor metastasis. Microarray analysis of metastatic ccRCC tissues identified that the lncRAN MRCCAT1 was found to be markedly overexpressed in metastatic ccRCC and correlated with an unfavorable prognosis in patients. lncRAN MRCCAT1 activates the p38 mitogen-activated protein kinase (MAPK) signaling cascade and drives ccRCC metastasis through epigenetic suppression of NPRC expression. This mechanism provides a new idea for prognostic assessment and targeted therapy of ccRCC. The findings from Nengwang Yu’s team also support this view that high expression of NPRC represented a better prognosis in sarcomatoid renal cell carcinoma. [41]. The study reported by Xingang Cui et al confirmed that NPRC functions as a critical biomarker indicative of highly differentiated, low-risk ccRCC. Its expression, specifically in conjunction with AQP1 (AQP1+NPRC+), defines the RCC-HD subtype, which is strongly associated with significantly improved patient survival outcomes compared to less differentiated, higher-risk subtypes. [42]. This positions NPRC expression as part of a molecular signature reflecting a favorable tumor biology and prognosis in ccRCC. In summary, these findings suggested that NPRC may function as a tumor suppressor gene in ccRCC. Furthermore, its significant value as a prognostic biomarker was clearly validated. However, the precise underlying molecular mechanisms require elucidation through future investigations.

#### Osteosarcoma (OS)

Li et al. [43] reported significantly lower than normal osteoblasts (hFOB1.19) across a range of OS cell cultures (e.g., MG63, Saos2, HOS, U2OS, etc.). Overexpression of NPRC was found to significantly inhibit OS cell proliferation through CCK-8 assay and flow cytometry evaluation revealed that NPRC overexpression led to arrest at the G1-phase of the cell cycle and enhanced programmed cell death. On the contrary, knockdown of NPRC increased cellular growth and expedited cell cycle progression (increased proportion of S-phase), and inhibited apoptosis. The researchers investigated the mechanism and found that NPRC acts via the downregulation of the PI3K/AKT pathway. Overexpression of NPRC significantly reduced the levels of PI3K and phosphorylated AKT. And NPRC was negatively regulated by POU2F1, whose overexpression reduced the expression of NPRC at both the transcript and protein levels. This in turn stimulated osteosarcoma cell proliferation by inhibiting NPRC. Therefore, the NPRC-POU2F1-PI3K/AKT axis, as a potential focal point for therapeutic approaches, provides the innovative approach for targeted intervention in OS. Research on the mechanisms of NPRC in OS remains limited, however, one study has reported NPRC was identified as one of the genes most significantly down-regulated in estrogen-dependent OS cells after long-term (3-month) exposure to bisphenols BPA, BPAF, and BPS [44]. The grouping with cardiovascular genes suggests its downregulation in this model might reflect a disruption of pathways important for cardiovascular function, but this doesn’t necessarily define its intrinsic role in the OS cells themselves.

#### Hepatocellular carcinoma (HCC)

Qian et al. [45] found that lncRNA FENDRR was significantly down-regulated in HCC tissues and cells (Huh-7, Hep3B, PLC/PRF/5), with a significant difference compared to normal liver tissues/cells (THLE-2). lncRNA FENDRR exerts oncogenic effects by targeting miR-362-5p, which promotes HCC progression by regulating the NPRC and p38-MAPK pathways to promote HCC progression. Overexpression of miR-362-5p negated the regulatory impact of FENDRR on HCC cell viability and apoptosis. In contrast, lncRNA FENDRR restored the previous state of influence of miR-362-5p on NPRC and the p38-MAPK signaling pathways. Thus, lncRNA FENDRR inhibits HCC cell proliferation and promotes apoptosis by adsorbing miR-362-5p, deregulating its inhibitory effect on the target gene NPRC, and inhibiting p38-MAPK pathway activation. This mechanism provides a new direction for molecularly targeted therapy of HCC.

## Roles and mechanism of the NPs/NPRs in antitumor therapy

### Antitumor therapeutic strategies targeting the NPs/NPRs

The NPs/NPRs system, beyond its well-established roles in cardiovascular and renal homeostasis [46], exhibits complex and context-dependent functions in cancer biology. While ANP demonstrates dual oncogenic and tumor-suppressive activities via NPRA. BNP and CNP similarly display multifaceted roles in tumor progression and suppression. Their effects are mediated through specific receptors (NPRA, NPRB, NPRC) and downstream signaling pathways, making them potential targets for innovative cancer therapies.

#### ANP

A 28-amino acid hormone primarily synthesized and secreted by atrial cardiomyocytes was initially studied for its role in regulating water-sodium balance and reducing blood pressure in cardiovascular and renal systems. ANP exerts its effects through specific receptors, with NPRA, being its primary bioactive receptor. NPRA is a transmembrane guanylyl cyclase receptor whose extracellular domain binds ANP, while its intracellular domain possesses guanylyl cyclase activity that catalyzes the conversion of GTP to cyclic GMP (cGMP), acting as a second messenger to activate downstream signaling pathways. A growing body of research indicates that the ANP/NPRA signaling pathway always contributes to the advancement of tumor development. Interestingly, in certain scenarios, ANP also exhibits antitumor effects via NPRA. Zhao [47] highlighted the dual nature of the ANP/NPRA axis in cancer, noting its ability to act both as an oncogene and a cancer-promoting factor. Ding et al. [48] proposed that dexamethasone was shown to upregulate ANP expression in cardiomyocytes. This regulatory effect implies that ANP may contribute to the inhibitory impact of dexamethasone on the proliferation of multiple myeloma cells. Other studies have revealed that the impact of ANP on tumor cell proliferation varies depending on its concentration. ANP activates the cGMP and PKG signaling cascades via the NPRA receptor, ultimately modulating ion channel activity and transcription factor expression. In their study, Zhang et al. [28] examined the levels of NPRA were assessed in gastric cancer cells (AGS) compared to normal gastric epithelial cells (GES-1). They observed that AGS cells expressed NPRA at high levels, while GES-1 cells did not express NPRA. Interestingly, they found that low doses of ANP (10⁻⁹ M) facilitated AGS cell growth via the cGMP-PKG-KCNQ1 signaling pathway. Conversely, high doses of ANP (10⁻⁶ M) curbed cell proliferation through a pathway independent of cGMP signaling. This intricate signaling pathway implies that NPRA could be a promising therapeutic target for cancer and encourages more research into its potentially opposing functions in tumor biology.

#### BNP

BNP primarily secreted by ventricular cardiomyocytes in response to volume/pressure overload, exists as a 32-amino acid active peptide and its inactive N-terminal fragment (NT-proBNP). Its role extends beyond heart failure diagnostics into cancer biology. Like ANP, BNP activates NPRA (guanylyl cyclase-A receptor), generating cGMP to regulate cell proliferation. However, BNP also binds the “clearance receptor” NPRC, which lacks guanylyl cyclase activity. NPRC activation triggers G-protein-dependent pathways, inhibiting adenylate cyclase or activating phospholipase C, thereby modulating ERK and PI3K/AKT pathways. Elevated plasma NT-proBNP levels are documented in multiple malignancies including lung cancer [49], multiple myeloma [50], neuroendocrine tumor [51], metastatic gastrointestinal stromal tumors (GIST) [52] and breast cancer [53–55]. While traditionally attributed to cardiac stress from chemotherapy or paraneoplastic effects, recent evidence suggests tumor cells directly secrete BNP. In prostate cancer, BNP/NT-proBNP elevation correlates with advanced stage and bone metastasis, potentially mediated by tumor-induced vascular shear stress [56]. High NT-proBNP predicts poor response to immunotherapy in lung cancer, possibly due to its association with systemic inflammation and immunosuppressive microenvironments [57]. NPRC also binds BNP, reducing its bioavailability and potentially dampening antitumor effects [58]. BNP’s effects are concentration-dependent. Low physiological levels may promote tumor growth via NPRC-mediated ERK activation, while supraphysiological doses induce apoptosis [59]. Elevated serum BNP correlates with reduced tumor burden in colorectal cancer patients, suggesting a protective role. However, its short half-life (<20 min) limits therapeutic use. Hybrid molecules (e.g., Isatin-BNP conjugates) are being explored to improve stability [60].

CNP exerts multifaceted antitumor effects primarily through NPRB-mediated vascular normalization and immunomodulation. Its engineered form, dCNP, overcomes pharmacokinetic barriers and demonstrates robust efficacy in preclinical solid tumor models, especially in combination with immunotherapy or chemotherapy [23].

#### CNP

A 22-amino acid peptide, primarily expressed by vascular endothelial cells [61–63]. Unlike ANP/BNP, it selectively binds to NPRB, a transmembrane guanylyl cyclase receptor, with minimal affinity for NPRA or the clearance receptor NPRC [64]. NPRB activation catalyzes intracellular cGMP production, triggering downstream effectors including cGMP-dependent protein kinase (PKG), phosphodiesterases, and ion channels. This pathway regulates vascular homeostasis, cell proliferation, and inflammation. NPRB is overexpressed in tumor-associated endothelial cells and stromal fibroblasts but minimally present in healthy tissues. This selective expression positions CNP/NPRB as a stromal-targeted axis in cancers like glioblastoma, osteosarcoma, and pancreatic ductal adenocarcinoma (PDAC). CNP’s most prominent antitumor action involves remodeling dysfunctional tumor vasculature. CNP enhances Angpt1 expression, stabilizing endothelial junctions by promoting VE-cadherin and occludin assembly. This reduces vascular leakage and suppresses plasma extravasation. CNP increases pericyte coverage of tumor vessels, decreasing tortuosity and improving blood perfusion. In murine models, this reduced intratumoral pressure by 40%, alleviating hypoxia. CNP downregulates collagen I, fibronectin, and fibroblast activation protein (FAP), reducing stromal stiffness and enhancing drug penetration. In 8 murine tumor models (e.g., liver/pancreatic/breast cancers), CNP derivatives normalized vessels, increased perfusion >2-fold, and suppressed hypoxia-inducible factor-1α (HIF-1α). High-dose CNP activates PKG, suppressing MAPK/ERK and PI3K/Akt pathways to inhibit proliferation in osteosarcoma and breast cancer cells. In melanoma models, CNP reduced lung metastases by 60% without affecting primary tumors, indicating selective inhibition of dissemination [23]. Native CNP has a short plasma half-life (3–5 min), limiting therapeutic utility [65]. The engineered derivative dCNP overcomes this [23]. Native CNP is acylated with C18 fatty acid chains, extending half-life to >24 h. Monotherapy with dCNP (0.3 mg/kg, subcutaneous) inhibited growth in colon/pancreatic/prostate cancers and extended survival in mice [23]. In metastasis models, dCNP reduced B16F10 melanoma lung nodules by 60%. dCNP enhances conventional and emerging cancer treatments: Doubled intratumoral cisplatin concentration in breast cancer models, improving tumor shrinkage by 50%. With anti-PD-1, dCNP increased response rates in renal cell carcinoma from 20% to 70%. Augmented CAR-T infiltration 3-fold in glioblastoma and prolonged survival by 40 days. Alleviated tumor hypoxia, increasing radiation sensitivity. NPRB expression as a predictor of dCNP response. Dual-modified CNP (e.g., glycosylation + acylation) to enhance solubility and tumor targeting [23].

### Regulatory mechanisms: interactions between the NPs/NPRs and tmour microenvironment

#### NPRA

NPRA promotes cancer development by affecting the tumor microenvironment (TME). The TME encompasses not only the tumor cells themselves but also various non-tumor components that interact with them. NPRA has been suggested to influence tumor development through several mechanisms, including modulating immune cell functions, fostering inflammation and cytokine release, activating cancer-associated fibroblasts (CAFs), facilitating new blood vessel development (neoangiogenesis) and playing a role in the regulation of metabolic processes [28]. NPRA promotes pro-angiogenic factors (VEGF, CXCR4), forming abnormal, leaky vessels that cause hypoxia and acidosis. They demonstrated that ANP/NPRA signaling may be a target for drug development against cancers and tissue injury repair [66]. Zhang et al. [67] explored the downstream signaling pathways initiated by the binding of ANP to its receptor NPRA. Their findings showed that inhibiting NPRA signaling, together with decreased TLR2 levels in dendritic cells, resulted in heightened release of IL-10 and TGF-β, as well as increased expression of SOCS3. These alterations resulted in an increased production of regulatory T cells, demonstrating that altering NPRA signaling within dendritic cells contributes to creating an immunosuppressive milieu, thereby enhancing immune tolerance. Jaya Mallela et al. [66] demonstrated that NPRA signaling is crucial for both baseline and inflammation-associated angiogenesis, processes that are fundamental to tumor development. In particular, this signaling pathway regulates the inflammatory environment within tumors by directing the recruitment of progenitor cells necessary for tumor maintenance and expansion. ANP has the potential to influence both inflammation and the tumor microenvironment, thereby affecting the progression of skin cancer. Its mechanism involves several key steps: ANP can suppress skin tumor growth and metastasis by inhibiting the NF-κB-mediated inflammatory response, decreasing mast cell infiltration, and reducing MMP activity. Additionally, ANP may lower NPRA expression through a cGMP-dependent feedback loop, which further modulates the tumor microenvironment and impacts skin cancer development [68]. The activity of NPRA has been shown to correlate with the levels of vascular endothelial growth factor (VEGF). A study carried out by Y Nakao et al. [31] demonstrated that NPRA can facilitate the spread and infiltration of tongue squamous cell carcinoma by enhancing tumor angiogenesis and lymphangiogenesis via VEGF modulation. Overall, NPRA not only plays a role in the cancer cells themselves, but also affects processes such as immune escape, angiogenesis and matrix remodeling by remodeling the TME. This suggests that therapeutic strategies targeting NPRA may need to integrate its multiple effects in different TMEs to develop more precise intervention programs.

#### NPRB

NPRB activation by dCNP promotes vascular normalization, suppressing hypoxia/fibroblast activation genes and TGF-β while enhancing pericyte coverage (30–50% increase), improving drug delivery and immune function [69]. dCNP-mediated NPRB activation increases intratumoral effector T cells, NK cells, and cDC1s with reduced exhaustion markers (PD-1/TIM-3). It upregulates T-cell activation genes and adhesion molecules/chemokines for enhanced immune cell trafficking. In PDAC models, dCNP synergizes with PD-1 inhibitors, tripling response rates [23].

#### NPRC

CNP reverses immunosuppression by reshaping immune cell dynamics. Increases infiltration of cytotoxic T lymphocytes (CTLs) and NK cells while depleting regulatory T cells (Tregs) and myeloid-derived suppressor cells (MDSCs). Downregulates exhaustion markers (PD-1, TIM-3) on CTLs and enhances IFN-γ/granzyme B production. Promotes type-1 dendritic cell (DC1) maturation, improving antigen presentation. These changes convert “cold” tumors (e.g., PDAC) into immunoresponsive “hot” tumors, potentiating checkpoint inhibitors [23].

### Regulatory mechanisms: cross-regulation of signaling pathways

**Fig. 2:**
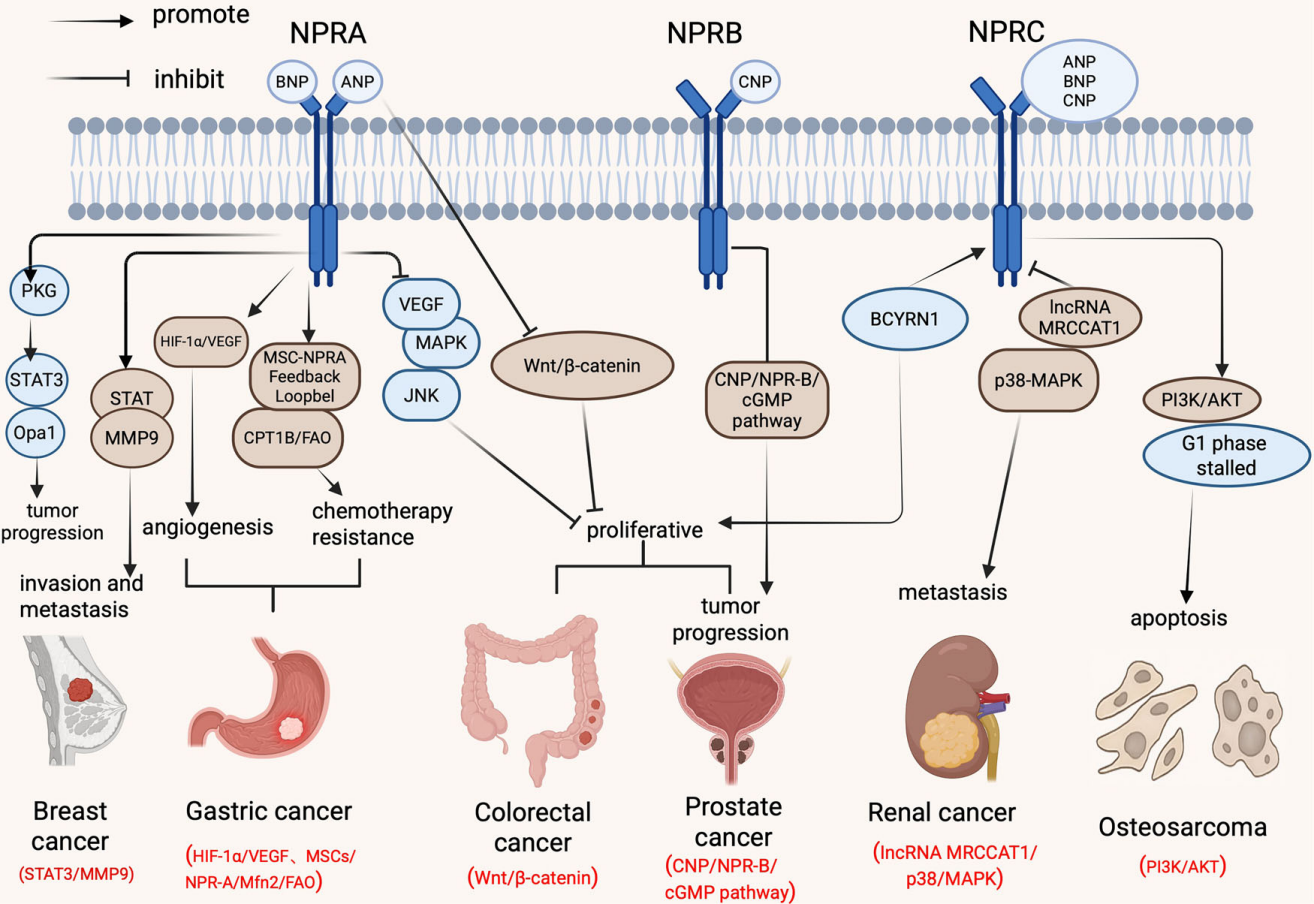
Overview the dual roles of NPR receptors across malignancies. NPRA is an oncogene. NPRA promotes proliferation, invasion, and angiogenesis in breast, gastric, and squamous cell carcinomas through activation of STAT3-MMP9, HIF-1α-VEGF pathways, and that its pro-carcinogenic effects are conserved across a wide range of tumors. NPRB changes in the level of its ligand, CNP, correlate with prostate cancer progression. NPRC exhibits dual functions: it promotes tumor progression by scavenging natriuretic peptides in prostate and colorectal cancers, whereas it exerts an oncogenic role through the suppression of the PI3K/AKT signaling pathway in osteosarcoma.

To elucidate the regulatory roles and signaling pathways of the NPs/NPRs system across diverse cancer types, we have systematically integrated their core mechanisms in Fig. 2. Nevertheless, this regulatory network exhibits intricate complexity involving multilevel mechanisms. The following sections will provide a detailed discussion on these aspects.

Kong et al. [70] demonstrated that NPRA expression and its associated signaling pathways play a vital part in facilitating the progression of tumor growth. Besides, antigen-induced lung inflammation was significantly reduced in NPRA-deficient mice. The absence of NPRA provided protective effects against lung, skin, and ovarian cancers in C57BL/6 mice. Their findings suggest that NPRA may contribute to tumor progression by modulating inflammatory responses, increasing resistance to programmed cell death, and inducing the development of new vasculature. Additionally, NPRA play a role in advancing cancer development in various malignancies, such as lung cancer, melanoma, and ovarian cancer. These results indicate that NPRA might serve as a prospective target for broad-spectrum anticancer therapies. The heart produces four distinct peptides, including vasodilator peptide, natriuretic peptide, and long-acting natriuretic peptide (LANP), not only have the physiological functions of blood volume regulation and diuresis, but also exhibit significant anticancer activity at their pharmacological concentrations. In vitro experiments have confirmed that these four peptide hormones can inhibit the proliferation of 97% of multiple forms of cancer cells, such as pancreatic, colon, prostate, mammary tissue, ovarian organs, renal, and glioblastoma [33, 71, 72]. In vivo experiments (thymus-free mouse model) verified that these peptide hormones eliminated 80% of pancreatic cancers, 86% of small-cell lung cancers, and 2/3 of breast cancers [73–75]. NPRA inhibited signaling pathways in cancer cells through multiple targets (e.g., inhibition of RAS-GTP activation (95%), MEK-1/2 (98%), and ERK-1/2 (96%), and inhibition of the RAS-MAPK pathway; blocked the oncogenic effects of β-Catenin (88%) by decreasing WNT3a (68%) and sFRP-3 (84%) via the WNT pathway; blocked tumor angiogenesis by dual inhibition of VEGF and VEGFR2 (89%); impaired cell viability and growth by decreasing AKT levels (64%); entered the cell nucleus to inhibit c-FOS (82%) and c-JUN (61%) and reduce AP-1 transcription factor activity; and selectively inhibit STAT3 (77-88%) without affecting STAT1 to block oncogene expression) [76]. These endogenous peptides have broad-spectrum anticancer activities and may reduce the limitations of single-target therapy through a multi-target mechanism, offering a conceptual foundation for creating new anticancer agents that act on multiple targets [76].

β-Catenin, known for its multifunctionality, resides on the inner surface of the cell’s plasma membrane [77], Secreted frizzled-related protein (SFRP) operates outside the cell to adjust Wnt signaling by either engaging directly with Wnt ligands or associating with frizzled (Fz) receptors [78]. Researchers have discovered that natriuretic peptides can influence Wnt signaling cascades both directly and indirectly. This modulation plays a key role in reducing the growth of human pancreatic cancer cells and also limits the proliferation of human colorectal adenocarcinoma cells [79]. Vesely’s research reported that ANP has the capability to directly suppress the Wnt/β-catenin signaling pathway. This inhibition results in decreased β-catenin expression within human pancreatic adenocarcinoma, colorectal adenocarcinoma, and renal carcinoma cells [80]. Although there have been no direct studies confirming the interaction of NPRs with the JNK pathway in cancer, ANP has been shown to inhibit the MAPK signaling pathway activated by vascular endothelial growth factor (VEGF) through interaction with its receptor NPRA. This inhibition involves key components such as JNK, ERK1/2, and p38, ultimately leading to the suppression of cell proliferation [81]. CNP stimulates the ERK1/2-MAPK pathway independently of cGMP signaling by interacting with the GC-B receptor. This process can influence the expression of pituitary-specific genes through the modulation of transcription factors, offering fresh insights into pituitary function and the mechanisms underlying tumor formation [82]. BNP enhances mitochondrial fusion by activating the NPRA-PKG pathway, which subsequently triggers the STAT3-Opa1 signaling cascade. This process leads to a reduction in mitochondrial ROS production [83]. ROS originating from mitochondria are crucial in driving tumor progression [84], therefore BNP may indirectly affect tumor progression through the STAT3-Opa1 signaling pathway. Overall, NPRs have been implicated in influencing cancer initiation, advancement, and therapeutic responses through their impact on oncogenic signaling cascades. However, further studies on the specific mechanisms of interaction between NPRs and these pathways in different cancer types are still needed.

## Conclusion

The NPR family exhibits distinct and often opposing roles in malignancy. NPRA predominantly functions as an oncogenic driver, with overexpression correlating with poor prognosis in breast, prostate, and gastric cancers. Its tumor-promoting effects are mediated through angiogenesis (VEGF upregulation), metastasis (MMP activation), stemness, chemoresistance (e.g., MSC-NPRA-FAO axis), and suppression of apoptosis. Inhibitors of NPRA signaling may have therapeutic potential in the treatment of cancers. Several NPRA antagonists are currently under investigation for other indications, and their repurposing for cancer therapy warrants further exploration. In contrast, NPRC consistently acts as a tumor suppressor across multiple cancers (e.g., ccRCC, OS, HCC, CRC), inhibiting proliferation, promoting apoptosis, and suppressing metastasis, often via pathways like PI3K/AKT or p38-MAPK. Targeting NPRC signaling through its inhibitors or enhancing natriuretic peptide activity with agonists could offer promising strategies for cancer therapy. Nonetheless, additional research is required to clarify NPRC’s precise involvement in cancer progression and to assess its viability as a target for therapeutic intervention. NPRB plays context-dependent roles but demonstrates therapeutic potential via engineered ligands (e.g., dCNP) that normalize tumor vasculature and remodel the stroma. Clinically, NPRA serves as a prognostic biomarker in gastric/esophageal cancers, while NPRC agonists (e.g., C-ANP4-23) and NPRB-targeting dCNP show promising antitumor efficacy in preclinical models. In conclusion, NPR family members can affect tumor progression by regulating fatty acid oxidation, metabolic reprogramming, and chemotherapy resistance, and their potential as prognostic markers (e.g., NPRA positively correlates with gastric cancer stage) and molecular imaging targets (NPRC-mediated imaging of prostate cancer) has been initially demonstrated. However, their dual functions underscore the need for context-specific therapeutic strategies. Future research should integrate multi-omics and clinical data to harness NPRs for precision oncology.

Future research needs to focus on the following key issues: 1 in-depth analysis of the signaling networks of different NPR family subtypes in tumors, especially their spatiotemporal specific activation mechanisms and epigenetic regulation; 2 development of highly selective targeting drugs, such as antagonists or NPRC agonists against NPRA, and exploration of their synergistic effects with traditional chemotherapy/immunotherapy; 3 establishment of precise therapeutic strategies based on molecular typing of the NPR family, such as targeting NPRA and NPRC agonists; 4 establishment of a new molecular modeling system for NPRA and NPRC. Precision therapeutic strategies based on molecular typing of NPR family, such as anti-angiogenic combination therapy for gastric cancer patients with upregulated levels of NPRA; 5 to validate the feasibility of NPR family as a liquid biopsy marker, such as detection of seminal plasma NT-proCNP level for dynamic monitoring of prostate cancer; 6 to focus on the functions of receptor heterodimerization and shear variants and elucidate their roles in tumor heterogeneity. With the development of nano-delivery systems and molecular imaging technologies, the integrated diagnostic and therapeutic solutions targeting the NPR family are expected to become a new breakthrough point in precision medicine for tumors, however, special attention must be paid to the variability of receptor functions in different tumor types to avoid generalizing therapeutic targets.

## References

[1] Wang X, Raulji P, Mohapatra SS, Patel R, Hellermann G, Kong X, et al. Natriuretic peptide receptor A as a novel target for prostate cancer. Mol Cancer. 2011;10:56. 21586128 10.1186/1476-4598-10-56 PMC3121714

[2] Mohapatra SS, Lockey RF, Vesely DL, Gower WR Jr. Natriuretic peptides and genesis of asthma: an emerging paradigm?. J Allergy Clin Immunol. 2004;114:520–6. 15356551 10.1016/j.jaci.2004.05.028

[3] Mohapatra SS. Role of natriuretic peptide signaling in modulating asthma and inflammation. Can J Physiol Pharmacol. 2007;85:754–9. 17823639 10.1139/Y07-066

[4] Sun Y, Eichelbaum EJ, Wang H, Vesely DL. Vessel dilator and kaliuretic peptide inhibit ERK 1/2 activation in human prostate cancer cells. Anticancer Res. 2006;26:3217–22. 17094432

[5] de Bold AJ, Borenstein HB, Veress AT, Sonnenberg H. A rapid and potent natriuretic response to intravenous injection of atrial myocardial extract in rats. Life Sci. 1981;28:89–94. 7219045 10.1016/0024-3205(81)90370-2

[6] Porter JG, Arfsten A, Palisi T, Scarborough RM, Lewicki JA, Seilhamer JJ. Cloning of a cDNA encoding porcine brain natriuretic peptide. J Biol Chem. 1989;264:6689–92. 2708334

[7] Sudoh T, Minamino N, Kangawa K, Matsuo H. C-type natriuretic peptide (CNP): a new member of natriuretic peptide family identified in porcine brain. Biochem Biophys Res Commun. 1990;168:863–70. 2139780 10.1016/0006-291x(90)92401-k

[8] Hirata Y. [Natriuretic peptide family]. Nihon Rinsho. 1993;51:1156–64. 8392634

[9] Moghtadaei M, Polina I, Rose RA. Electrophysiological effects of natriuretic peptides in the heart are mediated by multiple receptor subtypes. Prog Biophys Mol Biol. 2016;120:37–49. 26701223 10.1016/j.pbiomolbio.2015.12.001

[10] Xu M, Liu X, Li P, Yang Y, Zhang W, Zhao S, et al. Modified natriuretic peptides and their potential roles in cancer treatment. Biomed J. 2022;45:118–31. 34237455 10.1016/j.bj.2021.06.007 PMC9133251

[11] Takahashi Y, Nakayama T, Soma M, Izumi Y, Kanmatsuse K. Organization of the human natriuretic peptide receptor A gene. Biochem Biophys Res Commun. 1998;246:736–9. 9618281 10.1006/bbrc.1998.8693

[12] Gardner DG, Chen S, Glenn DJ, Grigsby CL. Molecular biology of the natriuretic peptide system: implications for physiology and hypertension. Hypertension. 2007;49:419–26. 17283251 10.1161/01.HYP.0000258532.07418.fa

[13] Chinkers M, Garbers DL, Chang MS, Lowe DG, Chin HM, Goeddel DV, Schulz S. A membrane form of guanylate cyclase is an atrial natriuretic peptide receptor. Nature. 1989;338:78–83. 2563900 10.1038/338078a0

[14] Misono KS, Philo JS, Arakawa T, Ogata CM, Qiu Y, Ogawa H, Young HS. Structure, signaling mechanism and regulation of the natriuretic peptide receptor guanylate cyclase. FEBS J. 2011;278:1818–29. 21375693 10.1111/j.1742-4658.2011.08083.x PMC3097287

[15] Rehemudula D, Nakayama T, Soma M, Takahashi Y, Uwabo J, Sato M, Izumi Y, Kanmatsuse K, Ozawa Y. Structure of the type B human natriuretic peptide receptor gene and association of a novel microsatellite polymorphism with essential hypertension. Circ Res. 1999;84:605–10. 10082481 10.1161/01.res.84.5.605

[16] Springer J, Azer J, Hua R, Robbins C, Adamczyk A, McBoyle S, Bissell MB, Rose RA. The natriuretic peptides BNP and CNP increase heart rate and electrical conduction by stimulating ionic currents in the sinoatrial node and atrial myocardium following activation of guanylyl cyclase-linked natriuretic peptide receptors. J Mol Cell Cardiol. 2012;52:1122–34. 22326431 10.1016/j.yjmcc.2012.01.018

[17] Fenrick R, McNicoll N, De Léan A. Glycosylation is critical for natriuretic peptide receptor-B function. Mol Cell Biochem. 1996;165:103–9. 8979258 10.1007/BF00229471

[18] Fenrick R, Bouchard N, McNicoll N, De Léan A. Glycosylation of asparagine 24 of the natriuretic peptide receptor-B is crucial for the formation of a competent ligand binding domain. Mol Cell Biochem. 1997;173:25–32. 9278251 10.1023/a:1006855522272

[19] Hirsch JR, Skutta N, Schlatter E. Signaling and distribution of NPR-Bi, the human splice form of the natriuretic peptide receptor type B. Am J Physiol Ren Physiol. 2003;285:F370–374. 10.1152/ajprenal.00049.2003 12709393

[20] Tamura N, Garbers DL. Regulation of the guanylyl cyclase-B receptor by alternative splicing. J Biol Chem. 2003;278:48880–9. 14514678 10.1074/jbc.M308680200

[21] Rahmutula D, Nakayama T, Soma M, Kosuge K, Aoi N, Izumi Y, Kanmatsuse K, Ozawa Y. Structure and polymorphisms of the human natriuretic peptide receptor C gene. Endocrine. 2002;17:85–90. 12041919 10.1385/ENDO:17:2:085

[22] Anand-Srivastava MB. Natriuretic peptide receptor-C signaling and regulation. Peptides. 2005;26:1044–59. 15911072 10.1016/j.peptides.2004.09.023

[23] Lu Z, Verginadis I, Kumazoe M, Castillo GM, Yao Y, Guerra RE, et al. Modified C-type natriuretic peptide normalizes tumor vasculature, reinvigorates antitumor immunity, and improves solid tumor therapies. Sci Transl Med. 2024;16:eadn0904. 39167664 10.1126/scitranslmed.adn0904 PMC11866103

[24] Qu J, Zhao X, Liu X, Sun Y, Wang J, Liu L, Wang J, Zhang J. Natriuretic peptide receptor a promotes breast cancer development by upregulating MMP9. Am J Cancer Res. 2019;9:1415–28. 31392078 PMC6682717

[25] Mao G, Zheng S, Li J, Liu X, Zhou Q, Cao J, et al. Glipizide combined with ANP suppresses breast cancer growth and metastasis by inhibiting angiogenesis through VEGF/VEGFR2 signaling. Anticancer Agents Med Chem. 2022;22:1735–41. 34515012 10.2174/1871520621666210910085733

[26] Li Z, Fan H, Cao J, Sun G, Sen W, Lv J, et al. Natriuretic peptide receptor a promotes gastric malignancy through angiogenesis process. Cell Death Dis. 2021;22:968. 10.1038/s41419-021-04266-7 PMC8528824 34671022

[27] Li Z, Wang J-W, Wang W-Z, Zhi X-F, Zhang Q, Li B-W, et al. Natriuretic peptide receptor A inhibition suppresses gastric cancer development through reactive oxygen species-mediated G2/M cell cycle arrest and cell death. Free Radic Biol Med. 2016;99:593–607. 27634171 10.1016/j.freeradbiomed.2016.08.019

[28] Zhang JIA, Zhao Z, Zu C, Hu H, Shen HUI, Zhang M, Wang J. Atrial natriuretic peptide modulates the proliferation of human gastric cancer cells via KCNQ1 expression. Oncol Lett. 2013;6:407–14. 24137337 10.3892/ol.2013.1425 PMC3789098

[29] Chen Z, Xu P, Wang X, Li Y, Yang J, Xia Y, et al. MSC-NPRA loop drives fatty acid oxidation to promote stemness and chemoresistance of gastric cancer. Cancer Lett. 2023;565. 10.1016/j.canlet.2023.216235 37209945

[30] Cao T, Wang S, Qian L, Wu C, Huang T, Wang Y, et al. NPRA promotes fatty acid metabolism and proliferation of gastric cancer cells by binding to PPARα. Transl Oncol. 2023;35:101734. 37418841 10.1016/j.tranon.2023.101734 PMC10345484

[31] Nakao Y, Yamada S, Yanamoto S, Tomioka T, Naruse T, Ikeda T, Kurita H, Umeda M. Natriuretic peptide receptor A is related to the expression of vascular endothelial growth factors A and C, and is associated with the invasion potential of tongue squamous cell carcinoma. Int J Oral Maxillofac Surg. 2017;46:1237–42. 28521969 10.1016/j.ijom.2017.04.022

[32] Zhao Z, Liu H, Yang Y, Sun K, Li M, Zhang J, Cai H, Wang J. Expression of natriuretic peptide receptor-A in esophageal squamous cell carcinomas and the relationship with tumor invasion and migration. World J Surg Oncol. 2014;12:154. 24885858 10.1186/1477-7819-12-154 PMC4038370

[33] Vesely BA, Alli AA, Song SJ, Gower WR Jr, Sanchez-Ramos J, Vesely DL. Four peptide hormones’ specific decrease (up to 97%) of human prostate carcinoma cells. Eur J Clin Investig. 2005;35:700–10. 16269020 10.1111/j.1365-2362.2005.01569.x

[34] Vesely BA, McAfee Q, Gower WR Jr, Vesely DL. Four peptides decrease the number of human pancreatic adenocarcinoma cells. Eur J Clin Investig. 2003;33:998–1005. 14636304 10.1046/j.1365-2362.2003.01262.x

[35] Schulz S. C-type natriuretic peptide and guanylyl cyclase B receptor. Peptides. 2005;26:1024–34. 15911070 10.1016/j.peptides.2004.08.027

[36] Lippert S, Iversen P, Brasso K, Goetze JP. C-type natriuretic peptide and its precursor: potential markers in human prostate cancer. Biomark Med. 2015;9:319–26. 25808436 10.2217/bmm.14.74

[37] Pressly ED, Pierce RA, Connal LA, Hawker CJ, Liu Y. Nanoparticle PET/CT imaging of natriuretic peptide clearance receptor in prostate cancer. Bioconjug Chem. 2013;24:196–204. 23272904 10.1021/bc300473x PMC3578065

[38] Gu L, Lu L, Zhou D, Liu Z. Long noncoding RNA BCYRN1 promotes the proliferation of colorectal cancer cells via up-regulating NPR3 expression. Cell Physiol Biochem. 2018;48:2337–49. 30114690 10.1159/000492649

[39] Martinez-Romero J, Bueno-Fortes S, Martín-Merino M, Ramirez de Molina A, De Las Rivas J. Survival marker genes of colorectal cancer derived from consistent transcriptomic profiling. BMC Genomics. 2018;19:857. 30537927 10.1186/s12864-018-5193-9 PMC6288855

[40] Li JK, Chen C, Liu JY, Shi JZ, Liu SP, Liu B, et al. Long noncoding RNA MRCCAT1 promotes metastasis of clear cell renal cell carcinoma via inhibiting NPR3 and activating p38-MAPK signaling. Mol Cancer. 2017;16:111. 28659173 10.1186/s12943-017-0681-0 PMC5490088

[41] Zuo Y, Fu S, Zhao Z, Li Z, Wu Y, Qi T, et al. Sarcomatoid-associated gene risk index for clear cell renal cell carcinoma. Front Genet. 2022;13:985641. 36159988 10.3389/fgene.2022.985641 PMC9493111

[42] Liu Y, Dong K, Yao Y, Lu B, Wang L, Ji G, et al. Construction and validation of renal cell carcinoma tumor cell differentiation-related prognostic classification (RCC-TCDC): an integrated bioinformatic analysis and clinical study. Ann Med. 2025;57:2490830. 40248945 10.1080/07853890.2025.2490830 PMC12010653

[43] Li S, Guo R, Peng Z, Quan B, Hu Y, Wang Y, Wang Y. NPR3, transcriptionally regulated by POU2F1, inhibits osteosarcoma cell growth through blocking the PI3K/AKT pathway. Cell Signal. 2021;86:110074. 34229087 10.1016/j.cellsig.2021.110074

[44] Fic A, Mlakar SJ, Juvan P, Mlakar V, Marc J, Dolenc MS, Broberg K, Mašič LP. Genome-wide gene expression profiling of low-dose, long-term exposure of human osteosarcoma cells to bisphenol A and its analogs bisphenols AF and S. Toxicol In Vitro. 2015;29:1060–9. 25912373 10.1016/j.tiv.2015.03.014

[45] Qian G, Jin X, Zhang L. LncRNA FENDRR upregulation promotes hepatic carcinoma cells apoptosis by targeting miR-362-5p Via NPR3 and p38-MAPK pathway. Cancer Biother Radiopharm. 2020;35:629–39. 32251605 10.1089/cbr.2019.3468

[46] Imura H, Nakao K, Itoh H. The natriuretic peptide system in the brain: implications in the central control of cardiovascular and neuroendocrine functions. Front Neuroendocrinol. 1992;13:217–49. 1334000

[47] Zhao Z, Zhang J, Li M, Yang Y, Sun K, Wang J. ANP-NPRA signaling pathway-a potential therapeutic target for the treatment of malignancy. Crit Rev Eukaryot Gene Expr. 2013;23:93–101. 23582032 10.1615/critreveukargeneexpr.2013006641

[48] Ding JH, Chang YS. Atrial natriuretic peptide: a possible mediator involved in dexamethasone’s inhibition of cell proliferation in multiple myeloma. Med Hypotheses. 2012;79:207–9. 22595807 10.1016/j.mehy.2012.04.037

[49] Aujollet N, Meyer M, Cailliod R, Combier F, Coignet Y, Campard S, Facy O, Bernard A, Girard C. High N-terminal pro-B-type natriuretic peptide: a biomarker of lung cancer?. Clin lung cancer. 2010;11:341–5. 20837460 10.3816/CLC.2010.n.043

[50] Pavo N, Cho A, Wurm R, Strunk G, Krauth M, Agis H, et al. N-terminal B-type natriuretic peptide (NT-proBNP) is associated with disease severity in multiple myeloma. Eur J Clin Investig. 2018;48:e12905. 10.1111/eci.12905 29417568

[51] Komarnicki P, Gut P, Musialkiewicz J, Cieslewicz M, Maciejewski A, Patel P, et al. NT-proBNP as a neuroendocrine tumor biomarker: beyond heart failure. Endocr Connect. 2023;12:230249. 10.1530/EC-23-0249 PMC10503219 37552533

[52] Perik PJ, Rikhof B, de Jong FA, Verweij J, Gietema JA, van der Graaf WT. Results of plasma N-terminal pro B-type natriuretic peptide and cardiac troponin monitoring in GIST patients do not support the existence of imatinib-induced cardiotoxicity. Ann Oncol. 2008;19:359–61. 17962203 10.1093/annonc/mdm468

[53] Silva FB, Romero WG, Carvalho AL, Borgo MV, Amorim MH, Gouvea SA, Abreu GR. Hormone therapy with tamoxifen reduces plasma levels of NT-B-type natriuretic peptide but does not change ventricular ejection fraction after chemotherapy in women with breast cancer. Braz J Med Biol Res. 2015;48:154–60. 25424369 10.1590/1414-431X20144189 PMC4321221

[54] Blancas I, Martín-Pérez FJ, Garrido JM, Rodríguez-Serrano F. NT-proBNP as predictor factor of cardiotoxicity during trastuzumab treatment in breast cancer patients. Breast. 2020;54:106–13. 32977298 10.1016/j.breast.2020.09.001 PMC7511727

[55] D’Errico MP, Grimaldi L, Petruzzelli MF, Gianicolo EA, Tramacere F, Monetti A, et al. N-terminal pro-B-type natriuretic peptide plasma levels as a potential biomarker for cardiac damage after radiotherapy in patients with left-sided breast cancer. Int J Radiat Oncol Biol Phys. 2012;82:e239–246. 21640499 10.1016/j.ijrobp.2011.03.058

[56] de Lemos JA, McGuire DK, Khera A, Das SR, Murphy SA, Omland T, Drazner MH. Screening the population for left ventricular hypertrophy and left ventricular systolic dysfunction using natriuretic peptides: results from the Dallas Heart Study. Am Heart J. 2009;157:746–753.e742. 19332205 10.1016/j.ahj.2008.12.017

[57] Li S, Du F, Zhang Y, Wang Q, Dou J, Meng X. Myocarditis prediction in locally advanced or metastatic lung cancer patients with cardiac parameters abnormalities undergoing immunotherapy: development and validation of a risk assessment model. BMC Cancer. 2025;25:541. 40133887 10.1186/s12885-025-13943-1 PMC11934563

[58] Meng QT, Liu XY, Liu XT, Liu J, Munanairi A, Barry DM, et al. BNP facilitates NMB-encoded histaminergic itch via NPRC-NMBR crosstalk. eLife. 2021;10:71689. 10.7554/eLife.71689 PMC8789279 34919054

[59] Khambata RS, Panayiotou CM, Hobbs AJ. Natriuretic peptide receptor-3 underpins the disparate regulation of endothelial and vascular smooth muscle cell proliferation by C-type natriuretic peptide. Br J Pharmacol. 2011;164:584–97. 21457229 10.1111/j.1476-5381.2011.01400.x PMC3178781

[60] Medvedev AE, Sandler M, Glover V. Interaction of isatin with type-a natriuretic peptide receptor: possible mechanism. Life Sci. 1998;62:2391–8. 9651105 10.1016/s0024-3205(98)00221-5

[61] Chauhan SD, Hobbs AJ, Ahluwalia A. C-type natriuretic peptide: new candidate for endothelium-derived hyperpolarising factor. Int J Biochem Cell Biol. 2004;36:1878–81. 15203101 10.1016/j.biocel.2003.09.009

[62] Pelisek J, Kuehnl A, Rolland PH, Mekkaoui C, Fuchs A, Walker GF, Ogris M, Wagner E, Nikol S. Functional analysis of genomic DNA, cDNA, and nucleotide sequence of the mature C-type natriuretic peptide gene in vascular cells.Arterioscler Thrombo Vasc Biol. 2004;24:1646–51. 10.1161/01.ATV.0000137387.78515.61 15231517

[63] Chen HH, Burnett JC Jr. C-type natriuretic peptide: the endothelial component of the natriuretic peptide system. J Cardiovasc Pharmacol. 1998;32 Suppl 3:S22–28. 9883743

[64] Del Ry S, Cabiati M, Vozzi F, Battolla B, Caselli C, Forini F, et al. Expression of C-type natriuretic peptide and its receptor NPR-B in cardiomyocytes. Peptides. 2011;32:1713–8. 21723350 10.1016/j.peptides.2011.06.014

[65] Moyes AJ, Hobbs AJ. C-type natriuretic peptide: a multifaceted paracrine regulator in the heart and vasculature. Int J Mol Sci. 2019;20:2281. 31072047 10.3390/ijms20092281 PMC6539462

[66] Mallela J, Ravi S, Jean Louis F, Mulaney B, Cheung M, Sree Garapati U, et al. Natriuretic peptide receptor A signaling regulates stem cell recruitment and angiogenesis: a model to study linkage between inflammation and tumorigenesis. Stem Cells. 2013;31:1321–9. 23533187 10.1002/stem.1376 PMC3982194

[67] Zhang W, Cao X, Chen D, Wang JW, Yang H, Wang W, et al. Plasmid-encoded NP73-102 modulates atrial natriuretic peptide receptor signaling and plays a critical role in inducing tolerogenic dendritic cells. Genet Vaccines Ther. 2011;9:3. 21219617 10.1186/1479-0556-9-3 PMC3025824

[68] Subramanian V, Vellaichamy E. Atrial natriuretic peptide (ANP) inhibits DMBA/croton oil induced skin tumor growth by modulating NF-κB, MMPs, and infiltrating mast cells in swiss albino mice. Eur J Pharmacol. 2014;740:388–97. 25058907 10.1016/j.ejphar.2014.07.024

[69] Yang J, Zhang Y, Xu X, Li J, Yuan F, Bo S, et al. Transforming growth factor-β is involved in maintaining oocyte meiotic arrest by promoting natriuretic peptide type C expression in mouse granulosa cells. Cell Death Dis. 2019;10:558. 31332164 10.1038/s41419-019-1797-5 PMC6646305

[70] Kong X, Wang X, Xu W, Behera S, Hellermann G, Kumar A, Lockey RF, Mohapatra S, Mohapatra SS. Natriuretic peptide receptor a as a novel anticancer target. Cancer Res. 2008;68:249–56. 18172317 10.1158/0008-5472.CAN-07-3086

[71] Saba SR, Vesely DL. Cardiac natriuretic peptides: hormones with anticancer effects that localize to nucleus, cytoplasm, endothelium, and fibroblasts of human cancers. Histol Histopathol. 2006;21:775–83. 16598676 10.14670/HH-21.775

[72] Vesely BA, Song S, Sanchez-Ramos J, Fitz SR, Solivan SM, Gower WR Jr, Vesely DL. Four peptide hormones decrease the number of human breast adenocarcinoma cells. Eur J Clin Investig. 2005;35:60–69. 15638821 10.1111/j.1365-2362.2005.01444.x

[73] Vesely DL, Eichelbaum EJ, Sun Y, Alli AA, Vesely BA, Luther SL, Gower WR Jr. Elimination of up to 80% of human pancreatic adenocarcinomas in athymic mice by cardiac hormones. In Vivo. 2007;21:445–51. 17591353

[74] Vesely DL, Vesely BA, Eichelbaum EJ, Sun Y, Alli AA, Gower WR Jr. Four cardiac hormones eliminate up to two-thirds of human breast cancers in athymic mice. In Vivo. 2007;21:973–8. 18210743

[75] Eichelbaum EJ, Sun Y, Alli AA, Gower WR Jr, Vesely DL. Cardiac and kidney hormones cure up to 86% of human small-cell lung cancers in mice. Eur J Clin Investig. 2008;38:562–70. 18717826 10.1111/j.1365-2362.2008.01978.x

[76] Vesely DL. Family of peptides synthesized in the human body have anticancer effects. Anticancer Res. 2014;34:1459–66. 24692673

[77] Mirabelli-Primdahl L, Gryfe R, Kim H, Millar A, Luceri C, Dale D, et al. Beta-catenin mutations are specific for colorectal carcinomas with microsatellite instability but occur in endometrial carcinomas irrespective of mutator pathway. Cancer Res. 1999;59:3346–51. 10416591

[78] Surana R, Sikka S, Cai W, Shin EM, Warrier SR, Tan HJ, et al. Secreted frizzled related proteins: Implications in cancers. Biochim Biophys Acta. 2014;1845:53–65. 24316024 10.1016/j.bbcan.2013.11.004

[79] Vesely DL. Cardiac hormones for the treatment of cancer. Endocr Relat Cancer. 2013;20:R113–125. 23533248 10.1530/ERC-13-0054

[80] Skelton WP, Pi GE, Vesely DL. Four cardiac hormones cause death of human cancer cells but not of healthy cells. Anticancer Res. 2011;31:395–402. 21378317

[81] Tripathi S, Pandey KN. Guanylyl cyclase/natriuretic peptide receptor-A signaling antagonizes the vascular endothelial growth factor-stimulated MAPKs and downstream effectors AP-1 and CREB in mouse mesangial cells. Mol Cell Biochem. 2012;368:47–59. 22610792 10.1007/s11010-012-1341-8 PMC3488346

[82] Jonas KC, Melrose T, Thompson IR, Baxter GF, Lipscomb VJ, Niessen SJ, et al. Natriuretic peptide activation of extracellular regulated kinase 1/2 (ERK1/2) pathway by particulate guanylyl cyclases in GH3 somatolactotropes. Cell Tissue Res. 2017;369:567–78. 28451751 10.1007/s00441-017-2624-x PMC5579180

[83] Chang P, Zhang X, Zhang J, Wang J, Wang X, Li M, Wang R, Yu J, Fu F. BNP protects against diabetic cardiomyopathy by promoting Opa1-mediated mitochondrial fusion via activating the PKG-STAT3 pathway. Redox Biol. 2023;62:102702. 37116257 10.1016/j.redox.2023.102702 PMC10165144

[84] Idelchik M, Begley U, Begley TJ, Melendez JA. Mitochondrial ROS control of cancer. Semin Cancer Biol. 2017;47:57–66. 28445781 10.1016/j.semcancer.2017.04.005 PMC5653465

